# Graphene Oxide/Polyethylenimine Aerogels for the Removal of Hg(II) from Water

**DOI:** 10.3390/gels8070452

**Published:** 2022-07-19

**Authors:** Alejandro Borrás, Bruno Henriques, Gil Gonçalves, Julio Fraile, Eduarda Pereira, Ana M. López-Periago, Concepción Domingo

**Affiliations:** 1 Materials Science Institute of Barcelona (ICMAB-CSIC), Campus UAB, 08193 Barcelona, Spain; aborras@icmab.es (A.B.); julio@icmab.es (J.F.); 2 LAQV-REQUIMTE, Department of Chemistry, University of Aveiro, 3810-193 Aveiro, Portugal; brunogalinho@ua.pt (B.H.); eduper@ua.pt (E.P.); 3 TEMA, Mechanical Engineering Department, University of Aveiro, 3810-193 Aveiro, Portugal; ggoncalves@ua.pt

**Keywords:** aerogel, supercritical CO_2_, Hg(II) sorption, graphene oxide, polyethylenimine

## Abstract

This article reports the synthesis of an aerogel involving reduced graphene oxide (rGO) and polyethylenimine (PEI), and describes its potential application as an effective sorbent to treat Hg(II) contaminated water. The rGO/PEI sorbent was synthetized using a supercritical CO_2_ method. N_2_ physisorption, electron microscopy, and elemental mapping were applied to visualize the meso/macroporous morphology formed by the supercritical drying. The advantages of the synthetized materials are highlighted with respect to the larger exposed GO surface for the PEI grafting of aerogels vs. cryogels, homogeneous distribution of the nitrogenated amino groups in the former and, finally, high Hg(II) sorption capacities. Sorption tests were performed starting from water solutions involving traces of Hg(II). Even though, the designed sorbent was able to eliminate almost all of the metal from the water phase, attaining in very short periods of time residual Hg(II) values as low as 3.5 µg L^−1^, which is close to the legal limits of drinking water of 1–2 µg L^−1^. rGO/PEI exhibited a remarkably high value for the maximum sorption capacity of Hg(II), in the order of 219 mg g^−1^. All of these factors indicate that the designed rGO/PEI aerogel can be considered as a promising candidate to treat Hg(II) contaminated wastewater.

## 1. Introduction

Hazardous heavy metal accumulation in surface water is continuously rising due to various anthropogenic activities, ranging from agriculture to transportation, and the direct disposal of industrial effluents in the rivers and to the sea, together with a developed irrational necessity for human comfort. The worldwide rapid decrease in water quality is currently recognized as a global environmental issue that has risks to public health, thus demanding urgent remediation solutions [1]. In particular, the Hg(II) contaminant is found worldwide, being one of the most toxic elements to aquatic organisms and human beings, even in minute traces [2]. Methods suitable to remove heavy metals from water include microfiltration and centrifugation after precipitation, flotation or flocculation, extraction and surface sorption, ion exchange, oxidation, and electrochemical treatments [3]. From them, sorption has emerged as the most convenient approach, principally due to the availability of a wide spectrum of re-usable low-cost sorbents with a high efficiency for removing metals present in water at low concentrations. Commonly used sorbents for heavy metal sorption are zeolites, porous carbons (activated carbon, carbon nanotubes and graphene derivatives), and metal oxides [4,5,6,7]. Recently, this area of research is evolving to the design of more sophisticated and complex sorbents [8,9]. Nevertheless, microporous activated carbon still remains the most cost-effective solution for water treatment, although this material is bound by important disadvantages related to pore blocking and slow contaminant diffusion. As a strong alternative, mesoporous graphene oxide (GO) sorbents are currently under scrutiny for water purification applications, although they still require important research efforts before optimization [10,11,12].

Heavy metal sorption studies on exfoliated graphene oxide (GO) started a decade ago, showing that this sorbent has extraordinary removal efficiency for some specific metals prone to establishing electrostatic interaction and/or complexation with the oxygenated functionalities on its surface [13]. However, the aggregation of exfoliated GO flakes in water cannot be circumvented during metal sorption, making the re-use of these sorbents problematic [14]. To attain improved performance, GO has been shaped into 3D-structured sorbents, for instance, in the form of aerogels [15,16,17,18]. These structures can be prepared by either freeze drying (lyophilization) [19,20] or by supercritical CO_2_ (scCO_2_) drying [21,22,23]. Lyophilization is a process of drying in which the solvent (e.g., water) is first frozen and then eliminated from the sample by sublimation. ScCO_2_ drying is a process in which the solvent (e.g., water) is first interchanged with a solvent miscible with compressed CO_2_ (e.g., ethanol) and then the solvent mixture is eliminated under supercritical conditions. In both cases, surface tension and capillary stress are avoided during drying. Materials commonly called aerogels or cryogels are obtained by using either supercritical or freeze drying, respectively [24,25,26]. The main disadvantages of the prepared 3D-structures are their extreme fragility and relatively poor mechanical properties, together with low structural stability when immersed in polar solvents, since they are obtained by non-covalent self-assembly [27]. A versatile strategy to increase the structural stability is hybridization with organic polymers [28]. The reinforced systems not only exhibit a great improvement in strength over the native compounds, but the polymer also confers different properties for new applications. Examples of intensely studied hybrid nanocomposites are GO/epoxy resin [29], GO/nanocellulose [30] GO/chitosan [31], and, particularly, GO/polyethylenimine (PEI) [20,32,33,34,35,36,37,38]. PEI is a polymer rich in electron donor amine groups, with metal chelating properties and a Lewis base character [39]. Under the area of interest of this work, the GO/PEI system has demonstrated high effectiveness for the removal of Hg(II) [20,40,41]. The main aim of this research is to advance the development of new graphene-based 3D sorbents for Hg(II) sorption with the sustainable scCO_2_ method. The synthetized hybrid aerogels can be thermally modified to aerogels of reduced GO (rGO) without damaging the other component, hence widening the spectrum of applications. The reduced form of GO, with a moderate number of oxygenated groups and still aromatic Lewis basicity, has also demonstrated a strong Hg(II) sorption capacity [42].

This work is part of an on-going study [24] analyzing the sorption of Hg(II) traces from very diluted water solutions (0.05 mg L^−1^) to reach values close to the limits of drinking water (e.g., 1 μg L^−1^ in Europe [43] and 2 μg L^−1^ according to the U.S. Environmental Protection Agency [44]). The synthetized rGO aerogels, either pristine or involving grafted PEI, were intensively characterized regarding their structure and composition. Furthermore, the aerogels were tested for their sorption capacity in water-spiked solutions containing Hg(II). The Hg(II) sorption kinetics and isotherms for the studied sorbents were estimated with the pseudo-first/pseudo-second order equations and the Langmuir/Freundlich models, respectively. The behavior of the supercritically synthetized rGO/PEI aerogel was evaluated in comparison to the reported similar GOPEI cryogels [20]. In particular, it has been demonstrated that the removal efficiency and the sorption capacity of the rGO/PEI aerogel prepared via the supercritical route are drastically improved with respect to the pristine rGO or similar GOPEI sorbents prepared using alternative methods (e.g., lyophilization).

## 2. Results and Discussion

### 2.1. Sorbents Synthesis and Structure

The nanocomposite aerogel sorbents studied in this work are based on rGO, although, to synthetize them through the supercritical route, the easily dispersible GO needs to be used as the starting material. GO aerogels were obtained by the non-covalent self-assembly of GO flakes during scCO_2_ drying, which refers to the construction of 3D structures through hydrogen bonding, involving the highly oxygenated surface (OH, -O-, COOH), and π–π interactions. The reduction process of GO to rGO was carried out to increase the 3D structural stability of the aerogel in water. Indeed, GO aerogels are prone to re-exfoliate in water, while rGO are not. A covalent self-assembly mechanism describes the formation of GO/PEI aerogels in which the occurrence of amide bonds, formed through the condensation of the primary amine groups in PEI with the carbonyl moieties and epoxy groups in GO, assures a stable 3D structure. GO/PEI aerogels were further reduced to rGO/PEI aerogels under thermal conditions mild enough not to affect the polymer.

**Figure 1:**
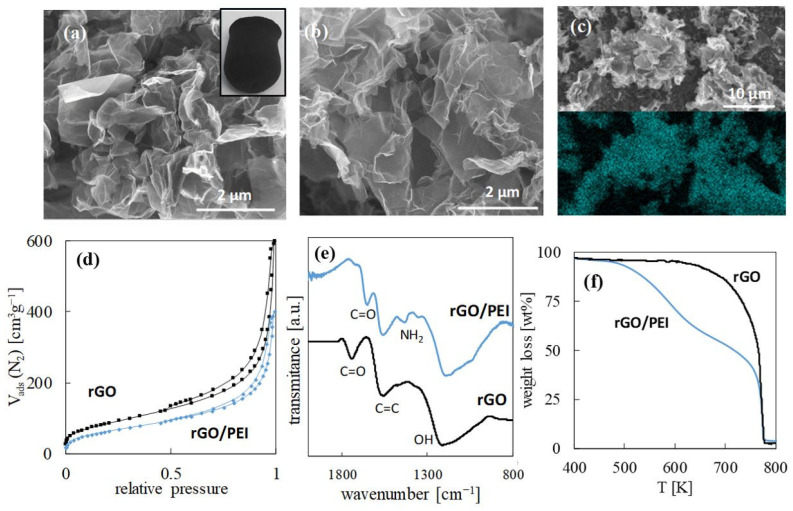
The characterization of the rGO and rGO/PEI sorbents: SEM of (**a**) rGO (the insight is an aerogel monolith) and (**b**) rGO/PEI; (**c**) EDS mapping of N_2_ for the rGO/PEI; (**d**) N_2_ adsorption/desorption isotherms; (**e**) FTIR-ATR spectra; and (**f**) TGA.

Scanning electron microscopy (SEM) images of the transversal section of the rGO and rGO/PEI monoliths displayed interconnected meso/macropores in a continuous network of highly exfoliated rGO flakes (Figure 1a,b). Essentially, a N mapping of the rGO/PEI surface, performed by energy dispersive spectroscopy (EDS) on a SEM image, showed that the polymer was homogeneously grafted on the surface (Figure 1c). N_2_ physisorption isotherms recorded for both sorbents (Figure 1d) can be described as type IV at low and medium relative pressure and type II at high relative pressure, which is characteristic of nanoporous structures with both meso- and macropores and a negligible contribution of microporosity. Hence, the structural aerogel-type mesoporosity of rGO was maintained after PEI incorporation as the polymer did not block the pores (Table 1).

The molecular structural analysis of rGO, performed by Fourier transform infrared spectroscopy with attenuated total reflection (FTIR-ATR, Figure 1e), indicates the presence of a large number of C=C bonds in the carbon network (band at 1550 cm^−1^) and a significant amount of the still remaining oxygenated groups shown by the band of OH at 1200 cm^−1^ and the signal of C=O at 1710 cm^−1^. The presence of PEI in the rGO/PEI sample was ascertained by a new signal appearing in the spectrum at 1430 cm^−1^ that was assigned to NH_2_ bending. Moreover, the carboxylic signal was observed to have shifted to 1650 cm^−1^ due to the bond established with the amino groups in PEI. The rest of the PEI signals were either coincident or overlapped by those of rGO. The TGA analysis of the rGO sample indicated that ca. a 6 wt% decrease occurred below 450 K, corresponding to the elimination of residual oxygen functionalities (Figure 1f). Hence, the used thermal reduction method (2 h a 473 K) was not harsh enough to totally eliminate the enormous amount of oxygenated functionalities present in the original GO aerogel (>25 wt%). Nevertheless, the applied reduction protocol was sufficient to transform the material from structurally unstable to stable in water by increasing the percentage of the aromatic structure, and thus the hydrophobic character. The composition of the rGO/PEI hybrid material was estimated by analyzing the different weight loss steps in the thermogravimetric analysis (TGA). The first weight decay of ca. 5 wt%, observed at temperatures < 450 K was assigned to the elimination of the remaining oxygenated groups. The second weight decay of ca. 42 wt%, observed between 500 and 700 K, was assigned to PEI decomposition and evaporation. The last weight decay at 780 K corresponded to rGO burning. The weight calculations indicate that the 1:1 wt GO/PEI precursor was altered to a 0.7:1 wt rGO/PEI nanocomposite during reduction.

### 2.2. Hg(II) Sorption Kinetics on rGO and rGO/PEI Sorbent

**Figure 2:**
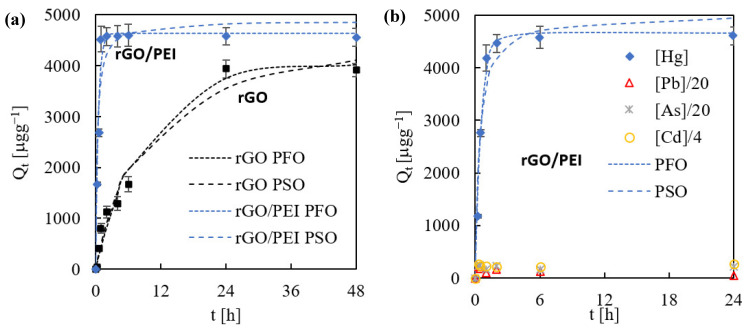
The experimental kinetic data points concerning the sorption of Hg(II) onto rGO/PEI over time and the fittings (dotted lines) of the PFO and PSO models for: (**a**) single metal, and (**b**) multicomponent solutions.

To evaluate the kinetic parameters, the sorption of Hg(II), at an initial concentration of 50 µg L^−1^ and pH 4.5, was evaluated at different time intervals up to reaching equilibrium. Constant concentrations of Hg(II) of ca. 50 µg L^−1^ were measured over the whole control experiments (e.g., without the addition of solid sorbent), indicating that potential losses due to sorption in glass walls or to volatilization could be considered as negligible. Different results were obtained for the rGO and rGO/PEI sorbents. rGO was very slow in sorbing Hg(II), attaining only 80 wt% removal after 48 h, while rGO/PEI was very fast, achieving near 95 wt% removal in the first hour. Collected experimental points were described using the pseudo-first (PFO) and pseudo-second (PSO) order kinetic models (Figure 2a).

For rGO, the experimental points were well-fitted by both the PFO and PSO models with an R^2^ of 0.97, although the Akaike’s information criterion (AIC) [45] for comparison gave a 63% probability of being PFO. For rGO/PEI, a better match was obtained by applying the PFO (R^2^ = 0.98) vs. PSO (R^2^ = 0.94) model, with an AIC probability of being PFO > 99%. The studied sorbents presented a very different k_1_ constant in the PFO model, with values of 0.11 and 2.0 h^−1^ for rGO and rGO/PEI, respectively (Table 2). Clearly, the rGO sorbent experienced stronger mass transfer limitations than rGO/PEI. Since both sorbents showed similar meso/macro porosity determined by low temperature N_2_ physisorption (Table 1), the external and intraparticle diffusion inside the monoliths was here not considered as the parameters controlling the uptake. For rGO, sorption of the sorbate on the active sites of the sorbent should be an important step controlling the reaction rate at intermediate and high uptake levels. In contrast, the high affinity of Hg(II) by amine-rich active sites in rGO/PEI sped up the sorption process at the interphase, giving an abrupt descent of metal concentration in the solution followed by a plateau. The residual Hg(II) concentration at equilibrium was 9.6 µg L^−1^ for rGO, and only 3.6 µg L^−1^ for rGO/PEI (Table 2). Hence, the rGO/PEI material synthetized in this work was able to reach residual values very close to the strict European and American standards in drinking water.

The sorption of Hg(II) in rGO/PEI was further analyzed in a multicomponent system involving, aside from Hg(II), also Pb(II), As(III), and Cd(II). The kinetic analysis indicated that the rGO/PEI was particularly selective for the sorption of Hg(II), since the concentration of the rest of the contaminants was practically invariable during the experiment, with R_E_ values close to zero (Figure 2b). Again, the PFO model was properly fit to the experimental data (R^2^ = 0.98), with a Hg(II) residual concentration of 3.4 µg L^−1^ at equilibrium, similar to that found in the single metal system (Table 2). The k_1_ constant was only slightly reduced from a value of 2 h^−1^ to 1.7 h^−1^ in multicomponent sorption, indicating low competition with other metals for sorption onto the active sites. 

### 2.3. Isotherm of rGO/PEI Sorbent for Hg(II) Sorption

**Figure 3:**
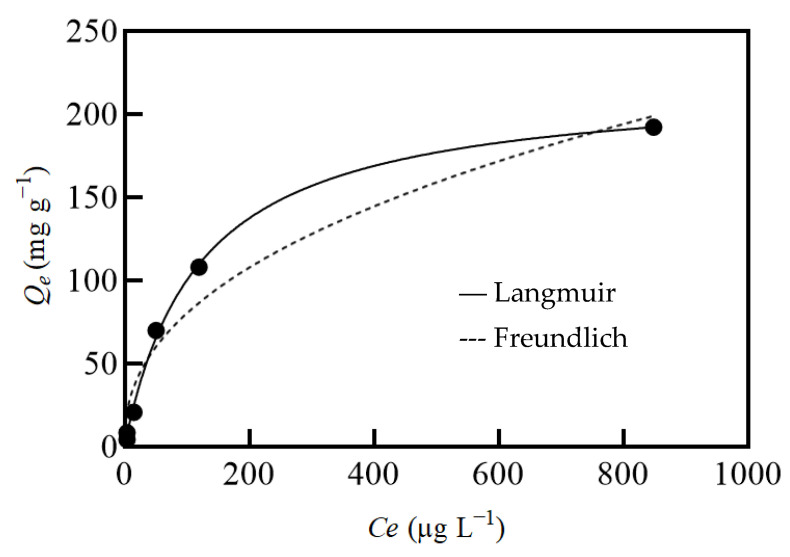
The Langmuir (straight line) and Freundlich (dotted line) isotherm models applied to the experimental data (dots) of Hg(II) sorption on rGO/PEI at different metal concentrations.

The equilibrium sorption capacity (Q_e_ mg g^−1^) of the hybrid sorbent was calculated by analyzing the influence of the different initial Hg(II) concentrations investigated in the range of 50–3000 µg L^−1^ at pH 4.5. Langmuir and Freundlich isotherms were used to model the experimental equilibrium data (Figure 3). Under the used experimental conditions, only the Langmuir model successfully described the data, with an R^2^ of 0.998, which indicates physical adsorption on homogeneous surfaces with only one type of binding mechanism. From the Langmuir equation, the maximum sorption capacity of the sorbent rGO/PEI (Q_e(max)_) for Hg(II) was estimated as 219 ± 13 µg g^−1^. The dimensionless separation factor R_L_, calculated from the constant of the Langmuir isotherm (0.0084) at a Hg(II) concentration of 50 µg L^−1^, obtained a value of 0.91, which is indicative of a highly favorable Hg(II) sorption process on the rGO/PEI surface.

### 2.4. Mechanism for Mercury Sorption by rGO/PEI

**Figure 4:**
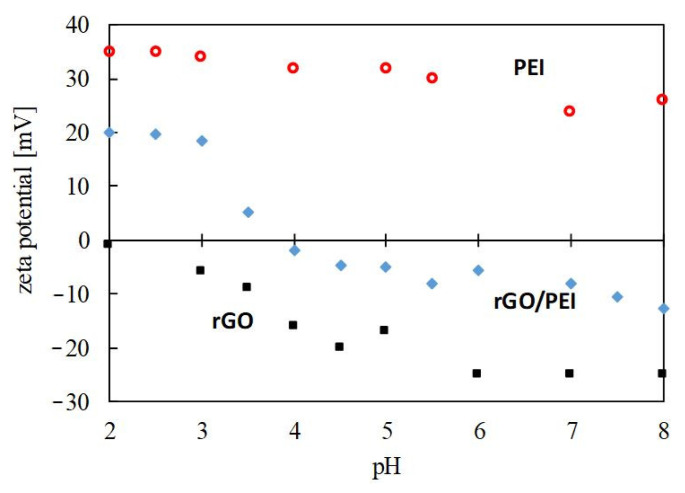
The zeta potential values measured for the studied sorbents in water as a function of pH.

The sorption of metals onto a sorbent is a surface phenomenon that strongly depends on two factors: first, the dominance of metal ion species at a particular solution pH and, second, the positive or negative surface charge of the sorbent at this pH [46,47]. In this work, a pH of 4.5 was chosen to carry out the Hg(II) sorption tests on the basis of previous studies that have shown, for similar systems involving GO and aminopolymers, that the removal of mercury increases substantially after a pH of 2, reaching a plateau at pH 4–5 [20,48]. Charged species on the support are suitable for complexation and electrostatic interactions with metals, while intermolecular and dipole–dipole or acid–base interactions are established between neutral species. The chemical species for Hg(II) in solution at different pH were calculated with the program Visual MINTEQ 3.1. At the used sorption pH of 4.5, the Hg(II) was mainly (98 wt%) in the form of neutral Hg(OH)_2_, with the remaining 2 wt% positively charged metal, Hg^2+^, and Hg(OH)^+^. A zeta potential study vs. pH was conducted to analyze the surface charge of bare rGO and hybrid rGO/PEI sorbents (Figure 4). 

**Figure 5:**
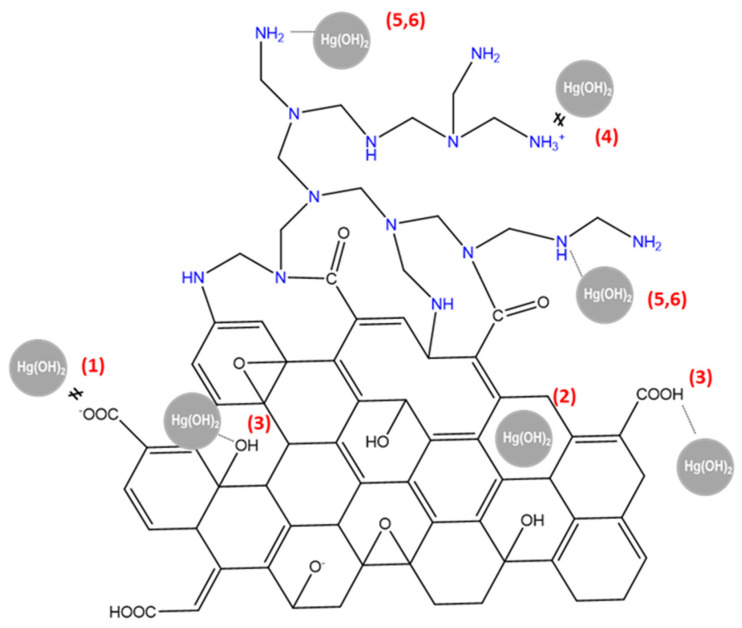
A schematic representation of the possible interactions represented in reactions (1–6).

On the basis of the surface charge analysis, the deduced possible interactions between the Hg(OH)_2_ and rGO or rGO/PEI sorbents are shown in reactions (1–6) and schematized in Figure 5.

- Electrostatic/complexation:

[rGO or rGO/PEI]-COO^−^/O^−^ + Hg(OH)_2_ → ≠ at pH 4.5 (1)

- Acid/basic aromatic π interaction:

[rGO or rGO/PEI]-C_6_H_6_ + Hg(OH)_2_ → [rGO or rGO/PEI]-C_6_H_6_···Hg(OH)_2_ (2)

- Hydrogen bonding:

[rGO or rGO/PEI]-COOH/OH + Hg(OH)_2_ → [rGO or rGO/PEI]-COOH/OH···Hg(OH)_2_ (3)

- Electrostatic/complexation/chelation:

[rGO/PEI]-NH^+^/NH_2_^+^/NH_3_^+^ + Hg(OH)_2_ → ≠ at pH 4.5 (4)

- Hydrogen bonding:

[rGO/PEI]-NH/NH_2_ + Hg(OH)_2_ → [rGO/PEI]-NH/NH_2_···Hg(OH)_2_ (5)

- Lewis base/acid coordination:

[rGO/PEI]-N/NH/NH_2_ + Hg(OH)_2_ → [rGO/PEI]-N/NH/NH_2_···Hg(OH)_2_ (6)

rGO displayed a highly negative zeta potential, ascribed to the still remaining high density of deprotonated hydroxyl and carboxyl functionalities on its surface. Electrostatic interactions between these oxygenated functionalities and the neutral Hg(OH)_2_ would not occur (reaction (1)), although it can be established with the residual positively charged cations. Moreover, rGO can formally act as a Lewis base in the sense that the aromatic rings in the structure can donate electrons to form a coordination bond. Hence, acid–basic interactions can be established between rGO and the soft acid bivalent mercury (reaction (2)). Finally, hydrogen bonding between the carboxyl and hydroxyl functionalities and neutral Hg(OH)_2_ is also a feasible interaction mechanism (reaction (3)). In contrast to rGO, the rGO/PEI sorbent displayed a positive or negative zeta potential value as a function of pH, with a zero-point charge observed at a pH of ca. 4. Above this pH, the charge of the sorbent was negative, given by an excess of oxygenated functionalities in rGO, and positive below the same, given by an excess of protonated amino-rich functionalities in PEI. In fact, the working pH of 4.5 is close to the point of zero charge, signifying that the surface charge of rGO/PEI should be relatively neutral, with the amine functionalities protonated only to a short extent, minimizing the electrostatic interactions (reaction (4)). Ahead of the interactions established with the rGO component, primary, secondary, and tertiary forms of amine, present in rGO/PEI, have a lone pair of electrons acting as a Lewis base prone for complexation with mercury (reaction (5)); and primary and secondary amines can be hydrogen bonded to neutral Hg(OH)_2_ (reaction (6)). For rGO, the binding mechanism is likely to be represented by the intermolecular interactions displayed in reactions (2,3), while for rGO/PEI, the amine-rich functionalities should be the main sorption sites (reactions (5,6)).

### 2.5. Design of Magnetic Sorbents

**Figure 6:**
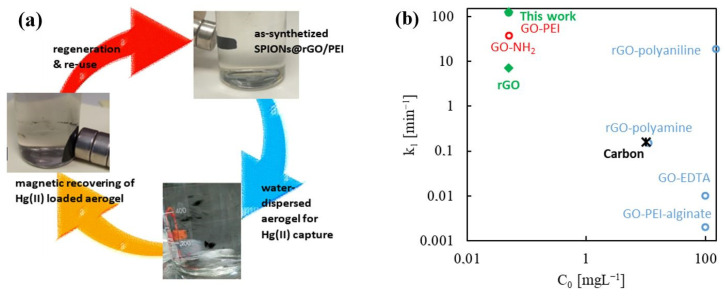
The behavior of the SPIONs@rGO/PEI aerogel synthetized for Hg(II) capture: (**a**) water behavior of the magnetic aerogel showing that an external magnet can be used to recover the loaded sorbent for re-use, and (**b**) the sorption of Hg(II) in a multicomponent solution onto rGO/PEI, either net or with deposited SPIONs, over time.

The water stability of rGO/PEI was studied through its regeneration and potential reuse in several Hg(II) sorption cycles. It is worth mentioning that the capacity of regeneration of this type of sorbent has already been demonstrated in a previous work for a similar GOPEI composite [20]. This type of material is easily regenerated by treating them with diluted HNO_3_ (2 v%). In this way, the pH of the medium is decreased and the cationic forms of mercury (Hg^2+^ and Hg(OH)^+^) become predominant. The affinity of these forms for the rGO/PEI, with a positive zeta potential at acid pH (Figure 4), would be minimal, thus allowing the desorption of the cationic metal. The pieces of monolith sorbent can then be recovered, washed with clean water, and reused. For a conventional aerogel, the sorbent must be separated by filtration. An alternative is the use of magnetic aerogels, in which the separation can be carried out with the aid of an external magnet [49,50,51]. The synthesis of magnetic aerogels involving superparamagnetic iron oxide nanoparticles (SPIONs) can be easily achieved using the supercritical route, as demonstrated previously by some of us [52]. The SPIONs were added in the desired percentage (ca. 20 wt%) to the precursor GO/PEI dispersion before scCO_2_ drying. The thermally reduced SPIONs@rGO/PEI aerogel, showing a magnetic character and structural water stability, provides magnetic susceptibility in front of an external field, which would facilitate the separation of the monolith pieces from the purified solution for reuse (Figure 6a). In this work, it was first verified that the behavior of rGO/PEI and SPIONs@rGO/PEI was similar with regard to the Hg(II) sorption capacity (Figure 6b), attaining for both materials an R_E_ of 95 wt% (C_0_ 50 µg L^−1^, doses 10 mg). In a second set of experiments, the SPIONs@rGO/PEI aerogel was used in two consecutive cycles of sorption/desorption. The performance of the sorbent for Hg(II) capture was maintained in the second cycle with an R_E_ value close to 94%, similar to the one of the as-synthetized aerogel (Figure 6b).

### 2.6. Contrasting Literature Data

**Figure 7:**
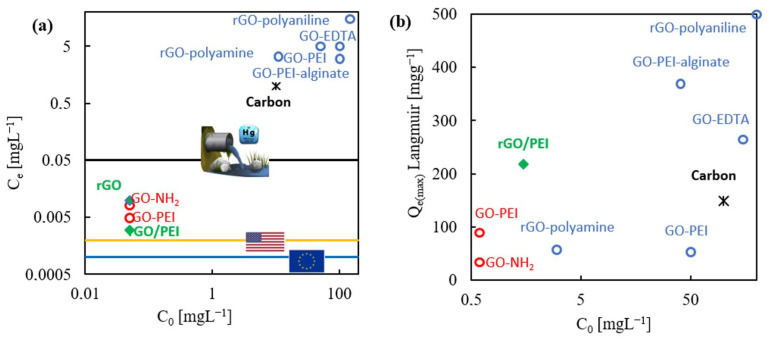
A comparison of the data obtained in this work and reported in the literature for several hybrids of GO and rGO sorbents involving nitrogen on the structure. Data are organized on the basis of the used initial concentration of Hg(II) (C_0_) represented vs.: (**a**) the residual equilibrium concentration in solution (C_e_), and (**b**) the maximum sorption capacity of the sorbent (Q_e(max)_). The literature information was extracted from references [20,41,54,55,56,57,58,59]. Data points in blue and red indicate tests performed at high and low initial concentrations of metal, respectively. Data points in green represent the results of this work.

The use of hybrid sorbents involving GO and nitrogen functionalities is a common practice for the sorption of Hg(II) from water. Sorption capacities ranging from 50 to more than 500 mg g^−1^ have been reported for these materials. However, most of the sorption tests have been performed at initial Hg(II) concentrations of 10–200 mg L^−1^, values that are drastically higher than the typical concentration of 0.05 mg L^−1^ used in this work. Different ranges of applied experimental conditions are represented in Figure 7a for several hybrid GO and rGO sorbents involving nitrogen on the structure [20,41,53,54,55,56,57,58]. In most of the preceding literature, the objective has been to reach residual Hg(II) values close to the permissible rates of discharge for industry (0.05 mg L^−1^), while the focus of this study was to start with concentrations in the range of the discharge rate and to reach values close to the limits of drinking water (e.g., 1 μg L^−1^ in Europe and 2 μg L^−1^ in the U.S. [43,44]) Heavy metals, in general, and Hg(II), in particular, are found in nature in the form of traces. Hence, the diluted concentration conditions used in this work to perform the sorption tests, from 0.05 to 3 mg L^−1^, are considered more realistic than the aforementioned literature values of 10 mg L^−1^ upward.

The relevant kinetic parameters chosen to consider a sorbent efficient are the residual concentration of metal in the water phase at equilibrium (Ce µg L^−1^) compared to the legal criteria, and, very importantly, the time required to achieve this equilibrium must be as short as possible. Invariably, for the tests performed at a high starting concentration of Hg(II), the residual Hg(II) concentration in the solution was higher than 1 mg L^−1^ (Figure 7a). Moreover, for only a few of these materials (e.g., rGO/polyamine [54]), the sorption was fast and the equilibrium was reached in less than one hour, although in this case, the attained maximum value of the sorption was only 58 mg g^−1^. In contrast, for most of the reported hybrid sorbents, the equilibrium was attained after several hours of contact time, as in the case of GO/PEI/alginate [42] or rGO/polyaniline [59], achieving in these cases high maximum sorption values > 300 mg g^−1^ (Figure 7b). 

It is remarkable that the rGO/PEI designed in this work was able to decrease the concentration of Hg(II) to only ca. 0.0035 mg L^−1^, very close to the values accepted for drinking water, in a very short period of time. When comparing the results of this work with those in the literature, it is important to consider that different sorption mechanisms may prevail in concentrated and diluted solutions, even when using similar solid sorbents. A high value for the initial Hg(II) concentration is often translated into metal excess in solution, which would create strong driving forces for metal diffusion in small pores and sorption in difficult to reach active sites. Moreover, processes of multilayer formation or heterogeneous precipitation for the metal (oxide, hydroxide) on the surface of the sorbent can be triggered under these conditions. As a consequence, higher maximum sorption values than the ones described for diluted systems [20] are found in concentrated Hg(II) solutions. Nevertheless, the maximum sorption capacity attained for the designed rGO/PEI aerogel was measured as 219 mg g^−1^ at concentrations of Hg(II) lower than 3 mg L^−1^. This value of Q_e(max)_ is high and close to those found on metal concentrated media, and it is almost double the value obtained for conventional industrial activated carbon, the latter in the order of 150 mg g^−1^ but obtained at an initial concentration of Hg(II) of 10 mg L^−1^.

**Figure 8:**
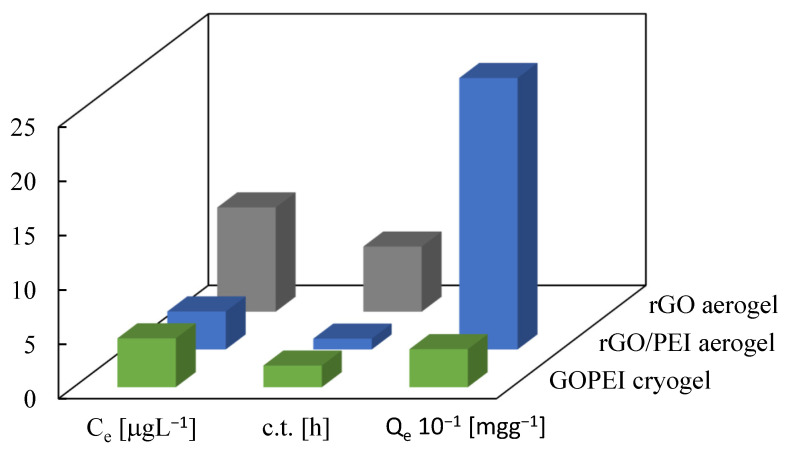
A diagram comparing the sorption data for a GOPEI cryogel (from [20]) and the rGO/PEI and rGO aerogels synthetized in this work.

The behavior of the rGO/PEI aerogel obtained in this work was compared to that of a GOPEI freeze-dried cryogel previously described by some of the authors in this work (Figure 8) [22]. The same hyperbranched high molecular weight PEI and similar GO:PEI ratio were used to build the monoliths in both cases, and sorption measurements were performed under similar experimental conditions (e.g., pH 4.5 and 50 µg L^−1^ of Hg(II) in the starting solution). First of all, it was observed that significantly higher values of maximum sorption capacity were obtained for the rGO/PEI aerogel (219 mg g^−1^) vs. the GOPEI cryogel (90 mg g^−1^). Additionally, noteworthy lower values of residual Hg(II) concentration in water were attained by using the rGO/PEI aerogel (3.5 µg L^−1^ in 1 h c.t.) instead of the GOPEI cryogel (4.5 µg L^−1^ in 2 h c.t.). 

The GOPEI cryogel described in the literature was obtained by lyophilization [20], while the rGO/PEI aerogel fabricated in this work was prepared by a scCO_2_ route. The morphology of water freeze-dried cryogels is frequently a replica of sublimated ice crystals, with pore sizes from a few to several hundreds of microns, very low density, but rather low specific surface area due to the loss of nanostructure in the pores, as constituent nanoentities are compressed by growing ice crystals [59]. When performing drying with scCO_2_, the resulting aerogels are open pore materials, with very high porosity and meso- and small macropores, resulting in high specific surface area materials. The SEM analysis of both sorbents showed a macroporous 3D network with largely interconnected channels, which is important to enable the fast diffusion of the sorbate inside the sorbent. As a consequence, the needed contact time for the equilibrium to be attained was similar in both cases. In contrast, the N_2_ adsorption/desorption isotherms of both products reported very different textural parameters. The GOPEI cryogel and rGO/PEI aerogel displayed values of the BJH mesopore volume in the order of 0.07 and 0.6 cm^3^ g^−1^, respectively. The low value of pore volume ascribed to mesopores (and small macropores) in the GOPEI cryogel is a consequence of the drying method, with the macropores prevailing in this material. Importantly, the surface area of 227 cm^3^ g^−1^ measured for the rGO/PEI aerogel decreased to 62 cm^3^ g^−1^ for the GOPEI cryogel, indicating that the latter is a less exfoliated system. This reduction in the surface area results in less available surface for PEI grafting, which should be highly packed on the GO surface suffering steric hindrance [60], and in a significant percentage of PEI blocked between the GO flakes. Hence, the GOPEI cryogel would present less available amine sorption sites for Hg(II) than the rGO/PEI aerogel.

## 3. Conclusions

This work presents the first example of cross-linked nanocomposites of amine functionalized GO (with PEI) in the form of an aerogel fabricated utilizing scCO_2_ technology. The synthetized macro/mesoporous aerogel was tested for Hg(II) sorption from trace metal contaminated water. Importantly, this sorbent removed up to 95 % of the Hg(II) in the water within less than 1 h, even with an initial metal concentration as low as 50 µg L^−1^, in which the driving force for sorption is low. The residual concentration of Hg(II) remaining in the contaminated water was in the order of 3.5 µg L^−1^, close to the limits for drinking water, while the maximum sorption capacity was in the order of 220 µg L^−1^, which is higher than most of the values obtained for the other sorbents reported in the literature. Actually, higher values of maximum sorption capacity and lower values of non-sorbed residual Hg(II) were obtained for the rGO/PEI aerogel when compared to a similar GOPEI cryogel measured under similar experimental conditions. In addition, the designed rGO/PEI aerogel sorbent was highly selective with regard to Hg(II), not losing the advantages of high removal capacity in very short contact times in multicomponent systems. Finally, magnetic rGO/PEI aerogels were also fabricated using the described scCO_2_ route, in which the magnetic character facilitates sorbent regeneration and reuse. Finally, the reached fast sorption kinetics are expected to greatly improve the economic efficiency of the process. 

## 4. Materials and Methods

### 4.1. Materials

GO nanosheets were supplied by Graphenea Inc. (Donostia-San Sebastian Guipúzcoa, Spain) as a stable dispersion in water with a concentration of 4 mg mL^−1^. Polyethylenimine (PEI, 50 *w*/*v*% in water, molecular weight ~750 k), and anhydrous absolute ethanol (EtOH) were all purchased from Sigma-Aldrich (St. Louis, MO, USA). Suprapur^®^ nitric acid (HNO_3_, 65 *w*/*v*% in water) was supplied by Supelco (Bellefonte, PA, USA). Ultrapure MilliQ^®^ water was used for dilution. Compressed CO_2_ (99.95 wt%) was delivered by Carburos Metálicos S.A. (Cornellà de Llobregat, Spain). Certified standard solutions from Merck of Hg(II), Pb(II), As(III), and Cd(II) (1000 mg L^−1^ in aqueous HNO_3_ 0.28 mol L^−1^) were used to contaminate ultrapure water up to the desired concentrations.

### 4.2. scCO*_2_* Synthesis of the Aerogels

Aerogels of GO and GO/PEI were prepared following an established scCO_2_ protocol designed to jellify and dry the flakes [21]. The basic precursor dispersion was a long-term stable colloidal suspension of GO in EtOH of a concentration of 4 mg L^−1^, prepared by following a multi-step water-to-ethanol exchange procedure. To formulate the GO/PEI precursor dispersion, measured volumes of PEI aqueous solution and GO suspension in water were mixed to obtain a mixture of a composition 5:1 wt for PEI:GO. The mixture was sonicated for 30 min and left under stirring at room temperature overnight. The water-to-ethanol solvent exchange protocol was then applied. During the solvent exchange, the excess of PEI non-bonded to the GO flakes was eliminated, ending in a dispersion of ca. 8 mg mL^−1^, which indicates a ratio of 1:1 wt for PEI:GO. For each material, three aliquots of 1 mL of the precursor suspension were added to three small vials, which were placed into a non-stirred high-pressure reactor of 100 mL. Then, liquid CO_2_ was flushed into the vessel to pressurize the system at ca. 6 MPa. The vessel was gently heated at 313 K and then pressurized up to 20 MPa. These experimental conditions were maintained for 48 h, a period after which the pressure was reduced to ambient by the slow release of the gas under isothermal conditions to avoid entering the two-phase region for the CO_2_. Finally, the reactor was allowed to cool down to room temperature and GO or GO/PEI monoliths were recovered in each case with a cylindrical shape. These aerogels were submitted to a soft thermal reduction process carried out under N_2_ flow at 473 K for 3 h, obtaining rGO and rGO/PEI reduced aerogels.

### 4.3. Solid State Characterization

The molecular arrangement was analyzed by Fourier transform infrared (FTIR) spectroscopy (Jasco 4700 Spectrophotometer, Jasco, Oklahoma City, OK, USA) using the attenuated total reflection (ATR) accessory and determining the main peaks in the 2000–800 cm^−1^ region. Thermogravimetric analysis (TGA, SDT 650, TA instruments, New Castle, DE, USA) was performed under oxygen flow (50 mL min^−1^) up to a temperature of 800 K increased in steps of 5 K min^−1^. Morphological features were examined using scanning electron microscopy (SEM, Quanta FEI 650FEG), also used to observe the atomic distribution of components by energy dispersive spectroscopy (EDS). The BET (Brunauer–Emmet–Teller) surface area (S_a_), the BJH (Barrett–Joyner–Halenda), and cumulative adsorption pore volume between 1.7–300 nm (V_p_) were determined by collecting N_2_ adsorption/desorption isotherms at 77 K (ASAP 2020, Micromeritics Inc., Norcross, GA, USA), after degassing the samples at 393 K for 20 h. The zeta potential values of the different bare components and nanocomposite were measured in aqueous suspensions (10 mg L^−1^) in the 2–8 pH range, adjusted with HNO_3_, using Malvern Zetasizer Nano ZS equipment (Malvern, UK).

### 4.4. Heavy Metal Batch Sorption Studies

The sorption of Hg(II) was studied using spiked waters prepared by diluting the certified solution of this metal with ultrapure water up to the desired concentration. The experiments were conducted at pH 4.5 with an initial Hg(II) concentration of 50 μg L^−1^, except for the ones that investigated the effect of metal concentration. Hg(II) sorption tests were also carried in multicontaminated water obtained by mixing volumes of the certified solutions of lead, arsenic, cadmium, and mercury to achieve a system with the following contaminant concentrations: [Pb(II)] = 1000 µg L^−1^, [As(III)] = 1000 µg L^−1^, [Cd(II)] = 200 µg L^−1^, and [Hg(II)] = 50 µg L^−1^, which correspond to the European maximum permissible values in wastewater discharges for these toxics. The pH was adjusted to the required value with HNO_3_ solution. To pre-equilibrate the systems, metal solutions were stirred overnight at 500 rpm. The sorption tests were performed in Schott bottles to which 10 mg of sorbent aerogel, broken in small pieces, were kept in contact with 1 L of contaminated water under stirring at 500 rpm. Aliquots of 5 mL were taken for analysis and immediately centrifuged at 5000 rpm to eliminate any residual solid sorbent. The supernatant liquid was acidified to pH ≤ 2 with HNO_3_ before metal concentration determination. The quantification of Hg(II) was performed by cold vapor atomic fluorescence spectroscopy using a CV-AFS PSA 10.025 Millennium Merlin Hg analyzer (P S Analytical, Orpington, UK) and SnCl_2_ (2 m/v% in HCl 10 v%) as a reducing agent. The quantification limit was 0.020 μg L^−1^ and the standard coefficient of variation among replicates was always <10%.

All assays were conducted in duplicate. A control experiment, corresponding to equally contaminated water, but in the absence of the solid sorbent, was every time run in parallel. The Hg(II) sorption kinetics were followed at pH 4.5 in solutions of 50 μg L^−1^ by collecting aliquots at defined periods of time (from 15 min up to 48 h). Sorption isotherms were measured, also at a pH of 4.5, in solutions of different initial concentrations of Hg(II) ranging from 50 to 3000 μg L^−1^, after a period of contact with the sorbent (10 mg L^−1^) that guarantees equilibrium conditions (e.g., 24–48 h). Inductively coupled plasma emission spectrometry (ICP-OES) was used to quantify Pb(II), As(III), and Cd(II) on a Jobin Yvon Activa M. An acceptable coefficient of variation among replicates of 10% was considered. The limits of quantification for Pb(II), As(II), and Cd(II) were 20, 20, and 2 µg L^−1^, respectively.

## Figures and Tables

**Table 1:** The textural properties of the studied sorbents including the BET (Brunauer–Emmet–Teller) surface area (S_a_) and the BJH (Barrett–Joyner–Halenda) pore volume (V_p_).

Sample	BET S_a_ (m^2^ g^−1^)	BJH V_p_ (cm^3^ g^−1^)
rGO	310	0.91
rGO/PEI	227	0.60

**Table 2:** The maximum removal percentage (R_E_) *, the contact time (c.t.) the sorbate–sorbent needed to reach equilibrium, and the PFO ** kinetic parameters (k_1_ and C_e_ ± standard deviations) obtained for the different sorbents regarding Hg(II) sorption in either a single or multicomponent solution.

Sample	Single Metal				Multicomponent			
	R_E_ (wt%)	c.t. (h)	k_1_ (h^−1^)	C_e_ (µg L^−1^)	R_E_ (wt%)	c.t. (h)	k_1_ (h^−1^)	C_e_ (µg L^−1^)
rGO	80	24	0.11 ± 0.03	9.6 ± 0.5	--	--	--	--
rGO/PEI	95	1	2.0 ± 0.04	3.6 ± 0.2	95	2	1.7 ± 0.09	3.4 ± 0.3

* $R_{E}=\frac{C_{0}-C_{e}}{C_{0}}\times 100$ C_0_ and C_e_ (µg L^−1^) are the initial and equilibrium concentrations of Hg(II). ** $\mathrm{PFO}:\;\frac{dQ_{t}}{d_{t}}=k_{1}\left(Q_{e}-Q_{t}\right),\;\mathrm{where}$ $Q_{t}=\frac{\left(C_{0}-C_{t}\right)v}{m}$ Q_e_ and Q_t_ (mg g^−1^) are the amounts of sorbed metal per gram of sorbent at equilibrium and time t [h]; k_1_ is the constant in PFO kinetic equation; v [L] is the volume of solution; m [mg] is the mass of the added solid sorbent.

## Data Availability

Not applicable.
